# Vulnerability and Burden of All-Cause Mortality Associated with Particulate Air Pollution during COVID-19 Pandemic: A Nationwide Observed Study in Italy

**DOI:** 10.3390/toxics9030056

**Published:** 2021-03-15

**Authors:** Tingting Ye, Rongbin Xu, Wenhua Yu, Zhaoyue Chen, Yuming Guo, Shanshan Li

**Affiliations:** 1 Climate, Air Quality Research Unit, School of Public Health and Preventive Medicine, Monash University, Melbourne, VIC 3004, Australia; tingting.ye@monash.edu (T.Y.); rongbin.xu@monash.edu (R.X.); wenhua.yu@monash.edu (W.Y.); 2 Barcelona Institute for Global Health (ISGlobal), 08003 Barcelona, Spain; zhaoyue.chen@isglobal.org; 3 Department of Epidemiology and Biostatistics, College of Public Health, Zhengzhou University, Zhengzhou 450001, China

**Keywords:** particulate matter, all-cause mortality, COVID-19, Italy

## Abstract

Background: Limited evidence is available on the health effects of particulate matter (PM including PM_2.5_ with an aerodynamic diameter ≤ 2.5 μm; PM_10_, ≤ 10 μm; PM_2.5–10_, 2.5–10 μm) during the pandemic of COVID-19 in Italy. The aims of the study were to examine the associations between all-cause mortality and PM in the pandemic period and compare them to the normal periods (2015–2019). Methods: We collected daily data regarding all-cause mortality (stratified by age and gender), and PM concentrations for 107 Italian provinces from 1 January 2015 to 31 May 2020. A time-stratified case-cross design with the distributed lag non-linear model was used to examine the association between PM and all-cause mortality. We also compared the counts and fractions of death attributable to PM in two periods. Results: Italy saw an increase in daily death counts while slight decreases in PM concentrations in pandemic period. Each 10 µg/m^3^ increase in PM was associated with much higher increase in daily all-cause mortality during the pandemic period compared to the same months during 2015–2019 (increased mortality rate: 7.24% (95%CI: 4.84%, 9.70%) versus 1.69% (95%CI: 1.12%, 2.25%) for PM_2.5_; 3.45% (95%CI: 2.58%, 4.34%) versus 1.11% (95%CI: 0.79%, 1.42%) for PM_10_; 4.25% (95%CI: 2.99%, 5.52%) versus 1.76% (95%CI: 1.14%, 2.38%) for PM_2.5–10_). The counts and fractions of deaths attributable to PM were higher in 2020 for PM_2.5_ (attributable death counts: 20,062 versus 3927 per year in 2015–2019; attributable fractions: 10.2% versus 2.4%), PM_10_ (15,112 versus 3999; 7.7% versus 2.5%), and PM_2.5–10_ (7193 versus 2303; 3.7% versus 1.4%). Conclusion: COVID-19 pandemic increased the vulnerability and excess cases of all-cause mortality associated with short-term exposure to PM_2.5_, PM_2.5–10_, and PM_10_ in Italy, despite a decline in air pollution level.

## 1. Introduction

The Coronavirus Disease 2019 (COVID-19) pandemic caused by the severe acute respiratory syndrome coronavirus 2 (SARS-CoV-2) virus [1] has substantially affected human society, including healthcare, economic structure, and social relationships. The measures and responses to control virus transmission can protect human health, but it also results in unprecedented side-effects. Even though the severe health impacts of the COVID-19 emergency remain the top priority, it is still unknown how the pandemic may affect the association between environmental exposure and health, notably the health impacts of air pollution, particularly for ambient particulate matter (PM) air pollutant, which is an important risk factor for cardiovascular and respiratory health outcomes [2,3].

The COVID-19 pandemic has observed a noteworthy decline in anthropogenic PM in many countries, such as China [4,5], Morocco [6], Malaysia [7], India [8], Brazil [9], the United States [10], and Spain [11], due to the reduced social activities and vehicle exhaust emission [5,12]. To control the COVID-19 outbreak, Italy was the first European country to impose a quarantine first in ten municipalities of the province of Lodi in Lombardy and neighboring municipalities in the northern region at around 21 February 2020, and then in early-March, the quarantine measures were expanded to the entire country [13]. Near the end of May 2020, the restrictions on movement were downgraded step-by-step. Alicandro et al. [14] suggested that Italy’s first wave of the COVID-19 pandemic has ended in May because there was no excess mortality, but COVID-19 deaths were probably over-registered. During the first wave of COVID-19 pandemic (March to May) in Italy, the severe limitation of people movements determined a significant reduction of PM_10_ and PM_2.5_ pollutants concentration mainly due to vehicular traffic, especially over hard-hit northern Italy (e.g., Milan [15] and Roma [16]). It is expected that reductions in PM could reduce burden of the air pollution-related diseases if the magnitude of mortality/morbidity risks associated with PM would not change. However, it is still unclear whether this hypothesis is correct because the exposure-response association could be changed due to the alteration of people’s behaviors and reorganized medical resources during the COVID-19 pandemic.

Previous assessments have focused on the effects of PM on the deaths related to COVID-19 [17,18,19,20]. However, the number of COVID-19 deaths in official reports may be significantly lower than the actual death toll due to the insufficient testing availability, especially during rapid growth phases of the epidemic [21]. In addition, indirect deaths from the pandemic may increase because of the limitation of health care resources, especially for the patients who were scheduled for surgery or treatment but were suspended due to the pandemic [22,23]. On the other hand, some susceptible populations are concerned about being infected when going to a hospital [24]. The overlap of vulnerable populations from COVID-19 and PM exposure makes the measurement of the pandemic impacts complex. Unlike the deaths attributed to COVID-19, all-cause mortality could be an accurate measurement to estimate the direct and indirect effects of the pandemic on deaths [25].

In this study, we aimed to quantify the association between short-term exposure to PM and all-cause mortality in Italy during the first wave of COVID-19 pandemic and compare it with that of the same months in 2015–2019 to identify the changes in PM-mortality association.

## 2. Materials and Methods

### 2.1. Study Area

Italy is a country consisting of a peninsula delimited by the Alps and surrounded by several islands, covering a total area of 301,340 km^2^. There are currently 107 provinces (second level constituent entities) in Italy, within 20 regions (first-level constituent entities). Its northern regions (Lombardia, Veneto, Piemonte, Emilia Romagna) together host 39% of the national population, and approximately one-half of the Italian GDP is produced there. Such a spatial concentration of economic activities involves the industrial manufacturing sectors to the largest extent, and the consequent high level of emissions is at least in part responsible for heavy pollution in the region [26]. During the COVID-19 pandemic, Italy has been one of the worst countries affected by the spreading of coronavirus, especially the northern regions [27].

### 2.2. Mortality Data

We collected daily all-cause mortality from Italian National Institute of Statistics (ISTAT) from 1 January 2015 to 31 May 2020. The mortality data cover 7357 municipalities in 107 provinces, representing 95.0% of Italy population since 1 January 2015. Daily counts of all-cause mortality were aggregated at province level and stratified by gender and age-specific groups (<65 years and ≥65 years).

### 2.3. Environmental Exposure Data

Air pollution data were downloaded from the European Environment Agency (EEA) air quality database (https://www.eea.europa.eu/data-and-maps/data/aqereporting-8,  accessed on 18 February 2021). The database includes hourly PM_2.5_, PM_10_, ozone (O_3_), nitrogen dioxide (NO_2_), sulfur dioxide (SO_2_), and carbon monoxide (CO). Our analysis method is based on a case-crossover design to compare the air pollution exposure in two different time periods (2015–2019 vs. 2020). To do this, we extracted air pollution data at station level, in which the beta attenuation monitoring (BAM) method was used to measure the levels of PM in Italy. BAM is a widely used air monitoring technique employing the absorption of beta radiation by solid particles extracted from air flow [28]. The 24-h average concentrations of PM_2.5_, PM_10_, NO_2_, SO_2_, and CO were calculated as daily concentration, while we used the maximum 8-h average concentrations of O_3_ as its daily value. Daily PM_2.5–10_ was calculated as the difference between 24-h average PM_10_ and 24-h average PM_2.5_ [29]. For both time-series analyses, daily air pollution, for each pollutant, in a province was calculated as the average of all central monitoring stations in that province. If a province only had one monitoring station, data from this station were used to represent the exposure level of this province.

To allow adjustment for the meteorological factors, we collected the ERA5 hourly surface (at 2 m above the land surface) ambient temperature and ambient dew point temperature at 0.1° × 0.1° spatial resolution from the ERA5-L and hourly data. Hourly data were averaged into daily values. We calibrated the collected temperature data with the observed meteorological data through random forest models (see Method S1 for detail), and then we linked the data to the centroid of each municipality based on longitude and latitude. We then calculated daily mean relative humidity from the calibrated ERA5 daily mean temperature and ERA5 daily mean dew point temperature, using the algorithm provided by the “humidity” R package [30]. Weather data at municipality level were aggregated into province level by averaging observations of all municipalities within the province.

### 2.4. Statistical Analysis

A time-stratified case-crossover design was used to examine the association between PM air pollution and all-cause mortality at national level. The design compares the air pollution exposure in the case period when events occurred with air pollution exposures in control periods to compare the differences in exposure, which might explain the differences in the daily number of cases. In this study, the province-level information was controlled by the time-stratified case-crossover design through matching case and control days by day of the week in the same calendar month, the same year, and in the same province. The Quasi-Poisson regression [31] allowing for over-dispersion was applied to perform time-stratified case-crossover design. To determine an appropriate lag time (i.e., the number of days between exposure and the estimated effect) for PM to be used in the main analyses, we compared a variety of lag days and choose all significant lags as the maximum lag day (Figure S3). A linear function was used for PM concentrations while a 3 degrees of freedom natural cubic spline was used for lags. Our initial analyses showed that significant mortality effects were observed in lag 0, 1, 2, and 3 days for PM_2.5_; lag 0, 1, and 2 days for PM_10_; and lag 0, 1 days for PM_2.5–10_. Therefore, we used cumulative effects along lag 0–3 days for PM_2.5_, lag 0–2 for PM_10_, and lag 0–1 for PM_2.5–10_ for subsequent analyses. We have controlled for potential nonlinear and lagged confounding effects of weather conditions, with 3 degrees of freedom natural cubic spline for 21-day moving averages [32,33] of daily mean temperature and daily mean relative humidity, respectively.

To compare the associations between PM air pollution and all-cause mortality during the COVID pandemic and pre-outbreak periods, we performed above analyses for 2020 COVID pandemic period (from 1 March to 31 May 2020) and the same months during 2015–2019, respectively. The analyses for PM_2.5_, PM_10_, and PM_2.5–10_ were performed separately to avoid their high collinearity. Fixed effect meta-regression was used to compare the magnitude of the mortality risks associated with PM air pollution in different time periods and sub-groups.

To estimate the burden of mortality attributable to PM, the attributable number deaths (AD) caused by PM were calculated every day and total AD was generated by summing the AD during the study period [34]. The corresponding attributable fractions (AF) of mortality were calculated by dividing the total AD by the death toll.

### 2.5. Sensitivity Analyses

Sensitivity analyses were performed to examine the robustness of the results. We tested the variation of the PM pollution-mortality association in the normal period by replacing the study period of 2015–2019 with every single year. To evaluate potential impacts of gaseous pollutants on the associations between PM air pollution and mortality, we also performed multi-pollutant models through adjusting for different combinations of NO_2_, CO, O_3_, and SO_2_.

All the analyses were performed by the R software (v. 3.6.1). The “dlnm” package was used to perform the distributed lag non-linear models for PM, describing simultaneously the linear relationship along air pollution and non-linear along lags; the “gnm” package was used to perform conditional Poisson regression [35]. The “mvmeta” package was used to perform meta-regression [36]. The relative risks (RRs) with 95% confidence intervals (CIs) per 10 µg/m^3^ change in PM concentration were reported. For all statistical tests, a *p*-value of 0.05 (two-tailed) was considered statistically significant.

## 3. Results

Daily air pollutants, meteorological variables, and all-cause death counts for each year in Italy are summarized in Table 1. There were 1,000,459 (51.9% females; 89.4% aged ≥65 years) all-cause deaths in the years from March to May during 2015 and 2020. The death counts in the pandemic period were significantly higher than the same months during 2015–2019 in subgroups. There were slight reductions in PM_10_, PM_2.5_, and PM_2.5–10_ concentrations over 2015–2020. The average (±SD) PM_2.5_ concentration during March and May reduced from 15.60 ± 10.23 μg/m^3^ in 2015 to 12.52 ± 7.57 μg/m^3^ in 2020, PM_10_ from 23.05 ± 12.25 μg/m^3^ to 20.54 ± 13.95 μg/m^3^, and PM_2.5–10_ from 8.86 ± 5.83 μg/m^3^ to 8.14 ± 7.43 μg/m^3^, with all the differences being statistically significant (*p* < 0.001).

**Figure 1:**
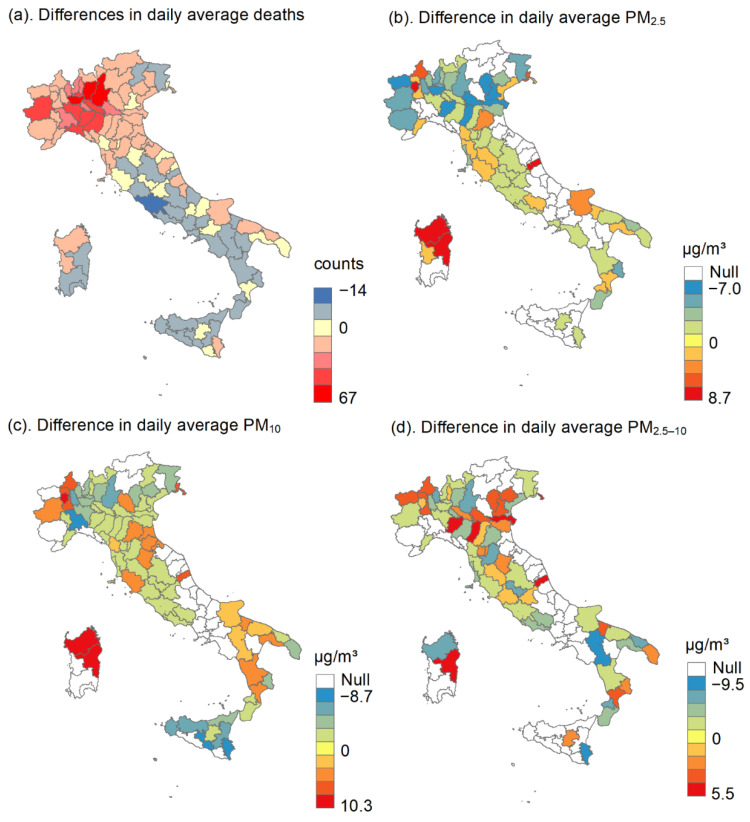
Difference in daily average deaths and the particulate matter (PM_2.5_ with an aerodynamic diameter ≤ 2.5 μm; PM_10_, ≤ 10 μm; PM_2.5–10_, 2.5–10 μm) in 107 Italian provinces during March and May. (**a**) Differences in daily average deaths, (**b**) Differences in daily average PM_2.5_, (**c**) Differences in daily average PM_10_, and (**d**) Differences in daily average PM_2.5–10_.

Figure 1 shows the spatial variation in all-cause death counts, PM_2.5_, PM_10_, and PM_2.5–10_ during March and May in 2020 and 2015–2019, respectively. The figure presents the difference by subtracting the average of 2015–2019 from the daily value in 2020. The northern region witnessed an increased number of deaths and declined PM concentrations in 2020.

**Figure 2:**
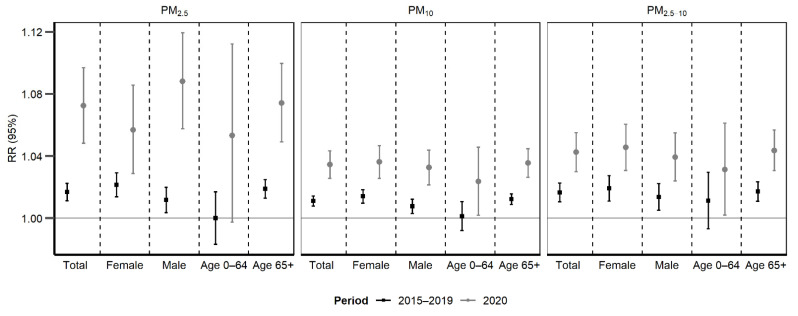
Cumulated relative risk (RR) of all-cause mortality and group-specific mortality associated with a 10 µg/m^3^ increase in the concentrations of particulate matter (PM_2.5_ with an aerodynamic diameter ≤ 2.5 μm; PM_10_, ≤ 10 μm; PM_2.5–10_, 2.5–10 μm).

Cumulative RRs along lag 0–3 days for PM_2.5_, lag 0–2 for PM_10_, and lag 0–1 for PM_2.5–10_ for all-cause mortality and group-specific mortality are shown in Figure 2. Each 10 µg/m^3^ increase in PM was associated with a much higher increase in daily all-cause mortality during the 2020 pandemic period compared to the same months during 2015–2019 (increased mortality risk: 7.24% (95% CI: 4.84, 9.70) versus 1.69% (95% CI: 1.12, 2.25) for PM_2.5_; 3.45% (95% CI: 2.58, 4.34) versus 1.11% (95% CI: 0.79, 1.42) for PM_10_; 4.25% (95% CI: 2.99, 5.52) versus 1.76% (95% CI: 1.14, 2.38) for PM_2.5–10_). All *p*-values for the difference of the 2020 pandemic period compared to the normal period during 2015–2019 were <0.001 (Table S1). Such disparity in the PM-mortality associations were consistent among different gender and age groups. The comparison of the risks between different periods and specific subgroups were in Table S1.

Table 2 shows the attributable mortality fractions and attributable deaths associated with PM_2.5_, PM_10_, and PM_2.5–10_ during March and May in 2020 and average values in 2015–2019. AFs and ADs were higher in 2020 than 2015–2019. We estimated that 10.21% (95% CI: 7.13, 13.31) of deaths were attributable to PM_2.5_ in the first three months of pandemic in 2020, whereas the average AF was only 2.44% (95% CI: 1.63, 3.23) in 2015–2019, and this disparity was consistent across all sex and age groups and was similar for PM_2.5–10_ and PM_10_. During March to May, 20,062, 15,112, and 7193 all-cause deaths were estimated to be attributable to PM_2.5_, PM_10_, and PM_2.5–10_ in 2020, which is approximately 5 times higher than the average values in 2015–2019.

Sensitivity results in Figure S4 consistently showed a stronger PM-mortality association during the 2020 pandemic period compared to the associations during the same months in each year of 2015–2019, despite the variations after adjusting for different gaseous pollutants. Likewise, when we used different lag days of PM (lag 0–4 and lag 0–5 for PM_2.5_, lag 0–3 and lag 0–4 for PM_10_, and lag 0–2 and lag 0–3 for PM_2.5–10_), the effects remained higher during the 2020 pandemic period than the same period in 2015–2019 (Figure S5).

## 4. Discussion

To the best of our knowledge, this the first study in the world to investigate the relationship between PM air pollution and daily all-cause mortality during the COVID-19 pandemic period. In this study, we examined the effects of PM (PM_2.5_, PM_10_, and PM_2.5–10_) on daily all-cause mortality in 107 Italian provinces and compared the mortality risks and mortality burdens associated with PM before and during the COVID-19 pandemic. The mortality risks (vulnerability) and burden associated with PM_2.5_, PM_10_, and PM_2.5–10_ in the pandemic were significantly higher than risks estimated in 2015–2019. People aged ≥ 65 years were consistently at higher risk than younger people in both pandemic period and normal periods. Exposure to PM air pollution has been identified as the risk factor for excess mortality [37,38]. Our results are in line with a previous multi-country epidemiological study [39], in which it observed a 0.65% (95% CI: 0.26%, 1.04%) increase in all-cause mortality risk per 10 μg/m^3^ increase at lag 0–1 of PM_10_ in 18 Italian cities during 2006–2015. Although the effect estimate is slightly lower than ours for PM_10_ at lag 0–2 (increased risk: 1.11% (95% CI: 0.79%, 1.42%)), such difference in the PM_10_-mortality associations could be ascribed to the heterogeneity in period and season, as PM-mortality association might be stronger during cold months that we chose [40].

In this study, we found that PM-attributed deaths during the COVID-19 pandemic in 2020 were 5 times higher than the same months during 2015–2019, despite a lower PM level. One potential explanation is that PM may contribute directly to the COVID-19 related deaths. Most current studies have reported a positive association between ambient PM_2.5_ or PM_10_ and COVID-19 deaths [26,41,42,43,44,45,46,47], although they were limited by the inaccurate official reports of COVID-19 deaths. A review study highlighted the potential role of PM in the spread of COVID-19, focusing on Italian cities in which correspondence between poor air quality and COVID-19 induced mortality was the starkest yet [48]. First, COVID-19 could have an air transmission [49,50] and atmospheric PM could create a suitable environment for transporting the virus at greater distances than those considered for close contact [51]. Second, PM has been shown to induce inflammation in lung cells [52] and exposure to PM could increase the susceptibility and severity of the COVID-19 patient symptoms [53].

However, the increase of PM-related mortality during the pandemic period could also be from non-COVID-19 causes, particularly for those vulnerable populations (e.g., elderly people, people with chronic diseases). For example, during the early days of the COVID-19 outbreak, the regional disparities in health-care resource availability and accessibility [54] could play an important role in the change of the vulnerability to PM related deaths. In March, though Italy’s health system has 3.2 hospital beds per 1000 people (as compared with 2.8 in the United States), it was over occupied to meet the needs of rapid surge of COVID-19 cases [55]. The health needs created by the coronavirus pandemic went well beyond the capacity of national health system, and diagnostic, therapeutic, and preventive interventions were scarce and rationed [54,56]. In the worst scenario, patients with PM-related diseases would die while waiting for needed resources (e.g., ICU, ventilator, and acute care) [57]. This hypothesis is supported by observations showing a significant decrease in hospitalization rates for acute coronary syndrome (13.3 admissions per day versus 18.0) [58] and acute myocardial infarction (a reduction of 52.1% in North Italy, 59.3% in Central Italy, and 52.1% in South Italy) [59] compared with the equivalent time in 2019 in Italy.

The interaction between SARS-CoV-2 infection and cardiovascular diseases could be another possible reason. Pierre et al. have examined the effects of PM on public health including increased risks of hospital admissions and mortality for respiratory and cardiovascular disease, and investigated that cardiovascular diseases are the main cause of mortality attributed to PM_10_ in Italy [60]. Current COVID-19 case reports show that patients with cardiovascular diseases may be more susceptible to SARS-CoV-2 infection. It can be seen not only that the number of COVID-19 patients with cardiovascular disease is large, but also that these patients have poor tolerance to severe pneumonia and are more likely to develop severe cases [61].

We found that the impacts of PM on all-cause mortality lasted longer during the pandemic period than those in normal time, and there was a trend that the mortality impacts strengthened rather than weakened with lag days elongated. This could be explained by delay or disruptions in routine and nonemergency medical care access and delivery caused by the pandemic and lockdown measures. In this scenario, the health conditions induced by PM exposure cannot be addressed timely; thus, they tended to become worse and worse as time went by. We also investigated the different effects between gender and age groups in the pandemic and normal periods. With regards to the age-stratified analysis, older people generally have a higher risk of PM-related death risk in both pandemic period and pre-pandemic periods, which was consistent with most previous studies [62,63]. Though the detailed reasons are still unclear, pre-existing cardiovascular and respiratory diseases are more prevalent in elderly people, and it may enable the elderly to be more susceptible to ambient PM air pollution.

There are some limitations to this study. First, like most case-crossover or time-series studies, we used province-level air pollution to represent the individual-level exposure, which is likely to cause random exposure assessment error, and thus, underestimate the PM-mortality associations [64]. However, since the same design was applied to the 2020 and 2015–2019 period, this error is not likely to affect our main findings. Second, we were not able to assess the actual indoor exposures to PM, which might be important because people would increase the indoor time during the lockdown period. We found evidence of the correlation between outdoor and indoor pollutant concentrations from previous studies in Italy or European cities. For example, the correlations between 24-h average indoor and outdoor PM_2.5_/PM_10_ concentrations were substantially high in Helsinki, Amsterdam, and Birmingham (correlation among cities ranged from 0.40 to 0.80 for PM_2.5_, 0.31 to 0.77 for PM_10_) [65]. Another study detected strong association (r = 0.74, *p* < 0.01) between indoor and outdoor PM observations across five European countries (Finland, Greece, Hungary, Italy, and the Netherlands) [66]. Wenjing et al. analyzed the published data to examine the health effects of indoor exposure to PM of outdoor origin, following an extensive review of the research in China, US, Europe (including Italy), and globally [67]. The findings suggest that indoor PM pollution of outdoor origin is a cause of considerable mortality, accounting for 81% to 89% of the total increase in mortality associated with exposure to outdoor PM pollution for the studied regions. Finally, we cannot exclude the COVID-19 deaths from our analyses because the daily data were not available at province level. Therefore, we were uncertain about whether the increase in PM-mortality association was due to COVID-19 deaths or deaths due to other causes.

Ambient PM exposures during the COVID-19 pandemic have important health implications. Even though the quarantine reduced air pollution level, the health cost is still significant given the increased vulnerability to ambient PM. In response to the pandemic, stricter regulation on air quality standard for ambient PM than pre-COVID time is needed. Secondly, our findings underscore the importance of giving close attention to specific vulnerable groups.

## 5. Conclusions

With a large nationwide data set covering 107 Italian provinces, we observed significantly increased impacts of PM on all-cause mortality during the pandemic period compared to pre-COVID-19 periods. This suggests the historical exposure-response relationship between PM and mortality may underestimate the health impacts of PM during the COVID-19 pandemic, although air pollution concentrations declined.

## Figures and Tables

**Table 1:** Descriptive statistics for temperature, relative humidity, and death counts during the first three months of COVID-19 pandemic in 2020 and the same months in 2015–2019.

Variables	2015	2016	2017	2018	2019	2015–2019	2020	*p* Value
Temperature (°C)	12.32 (4.51)	12.20 (4.14)	13.01 (4.07)	12.75 (5.18)	11.48 (3.1)	12.35 (4.29)	12.52 (4.40)	<0.001
RH (%)	67.62 (11.45)	69.12 (10.43)	65.64 (10.96)	72.76 (9.43)	68.85 (12.07)	68.80 (11.15)	65.41 (12.08)	<0.001
PM_2.5_ (μg/m^3^)	15.60 (10.23)	12.60 (7.71)	13.59 (9.50)	13.38 (8.49)	11.77 (8.02)	13.55 (9.01)	12.52 (7.57)	<0.001
PM_10_ (μg/m^3^)	23.05 (12.25)	20.42 (13.46)	20.73 (12.45)	22.66 (12.21)	19.71 (12.95)	21.40 (12.7)	20.54 (13.95)	<0.001
PM_2.5–10_ (μg/m^3^)	8.86 (5.83)	8.24 (6.78)	8.46 (5.66)	8.48 (6.16)	8.49 (6.07)	8.51 (6.83)	8.14 (7.43)	<0.001
NO_2_ (μg/m^3^)	21.54 (11.09)	19.88 (9.87)	20.31 (10.84)	19.36 (10.61)	17.96 (9.90)	19.91 (10.56)	11.96 (7.19)	<0.001
CO (mg/m_3_)	0.49 (0.29)	0.44 (0.25)	0.41 (0.22)	0.45 (0.24)	0.40 (0.17)	0.44 (0.24)	0.38 (0.24)	<0.001
O_3_ (μg/m^3^)	92.93 (18.97)	87.55 (18.94)	94.95 (18.45)	85.80 (20.71)	83.36 (20.75)	89.15 (20.00)	85.13 (22.66)	<0.001
SO_2_ (μg/m^3^)	3.35 (2.70)	2.89 (2.39)	2.83 (2.46)	2.63 (2.31)	2.82 (3.49)	2.91 (2.66)	2.46 (2.89)	<0.001
Daily Average deaths counts								
Age < 65 years	200 (17)	192 (15)	190 (15)	192 (17)	187(17)	192 (17)	196 (37)	0.097
Age ≥ 65 years	1590 (150)	1523 (104)	1544 (82)	1548 (154)	1573 (125)	1555 (128)	1939 (556)	<0.001
Female	941 (93)	887 (66)	903 (53)	906 (93)	918 (80)	911 (80)	1093 (271)	<0.001
Male	849 (70)	828 (50)	831 (46)	835 (73)	842 (64)	837 (62)	1043 (325)	<0.001
Total	1790 (157)	1715 (109)	1734 (88)	1740 (161)	1759 (135)	1748 (135)	2136 (590)	<0.001

Mean and SD (Standard deviation) were presented; *p*-Value was calculated by independent Two-Sample *t*-Test comparing observations in 2020 versus all observations in 2015–2019.

**Table 2:** Attributable fractions and attributable counts of all-cause mortality due to PM_2.5_, PM_10_, and PM_2.5–10_ during March to May in 2020 and 2015–2019, respectively.

PM	Subgroups	Attributable Mortality Fractions (%)		Attributable Deaths	
		2020	2015–2019	2020	2015–2019
PM_2.5_	Total	10.21 (7.13, 13.31)	2.44 (1.63, 3.23)	20,062 (13,811, 25,862)	3927 (2693, 5171)
	Female	8.05 (4.15, 11.60)	3.11 (1.95, 4.22)	8094 (4129, 11688)	2605 (1680, 3560)
	Male	12.36 (8.26, 16.29)	1.70 (0.47, 2.87)	11,860 (8009, 15371)	1308 (465, 2182)
	Age < 65 years	7.42 (−0.41, 14.32)	0.01 (−2.54, 2.43)	1340 (−86, 2685)	1 (−474, 454)
	Age ≥ 65 years	10.47 (7.34, 13.76)	2.73 (1.85, 3.57)	18,675 (12,494, 23,935)	3902 (2691, 5006)
PM_10_	Total	7.69 (5.82, 9.59)	2.49 (1.80, 3.19)	15,112 (11,381, 18,574)	3999 (2886, 5111)
	Female	7.97 (5.74, 10.09)	3.16 (2.21, 4.13)	8013 (5793, 10150)	2652 (1844, 3449)
	Male	7.38 (4.99, 9.96)	1.73 (0.72, 2.79)	7081 (4758, 9233)	1334 (542, 2103)
	Age < 65 years	5.21 (0.72, 9.78)	0.3 (−1.87, 2.33)	942 (147, 1721)	53 (−345, 418)
	Age ≥ 65 years	7.93 (6.08, 9.85)	2.75 (2.1, 3.43)	14,144 (10,859, 17,330)	3932 (2883, 4948)
PM_2.5–10_	Total	3.66 (2.67, 4.67)	1.43 (0.88, 1.94)	7193 (5163, 9144)	2303 (1494, 3119)
	Female	3.88 (2.65, 5.04)	1.66 (0.99, 2.39)	3905 (2734, 5073)	1389 (819, 1991)
	Male	3.43 (2.13, 4.69)	1.18 (0.39, 1.89)	3293 (2085, 4452)	910 (296, 1471)
	Age < 65 years	2.70 (0.15, 4.93)	0.99 (−0.62, 2.46)	488 (59, 934)	175 (−102, 436)
	Age ≥ 65 years	3.76 (2.67, 4.82)	1.48 (0.97, 1.98)	6707 (4705, 8451)	2123 (1375, 2861)

To be able to compare with the attributed deaths in 2020 (single year), we reported annual average attributed deaths in 2015–2019 (i.e., dividing the cumulated value by 5 years).

## Supplementary Materials

The following are available online at https://www.mdpi.com/2305-6304/9/3/56/s1, Table S1: Estimation of the differences of increased relative risk (RR) between different time periods and subgroups, Method S1: Calibration of ERA5 temperature data against weather observations, Figure S1: Distribution of weather stations that used to calibrate the ERA5 daily temperature and dew point temperature (see Method S1), Figure S2: 10-fold cross-validation of the random forest model that using ERA5 daily mean temperature and dew point temperature and other covariates to predict the daily mean temperature and dew point temperature observed by weather stations. (A) daily mean temperature, and (B) daily mean dew point temperature (see Method S1), Figure S3: RR (mean and 95%CI) of all-cause mortality associated with per 10 μg/m^3^ increase in PM concentrations in single-pollutant models on different lag days of PM_2.5_, PM_10_ and PM_2.5–10_, Figure S4: Cumulated RR (mean and 95%CI) of all-cause mortality associated with per 10 μg/m^3^ increase in PM concentrations in single- and multi-pollutant models, Figure S5: Cumulative RR (mean and 95%CI) of all-cause mortality associated with per 10 μg/m^3^ increase in PM concentrations in single-pollutant models on different lag days of PM_2.5_, PM_10_ and PM_2.5–10_.

## References

[1] Huang C., Wang Y., Li X., Ren L., Zhao J., Hu Y., Zhang L., Fan G., Xu J., Gu X. (2020). Clinical features of patients infected with 2019 novel coronavirus in Wuhan, China. Lancet.

[2] Burnett R., Chen H., Szyszkowicz M., Fann N., Hubbell B., Pope C.A., Apte J.S., Brauer M., Cohen A., Weichenthal S. (2018). Global estimates of mortality associated with long-term exposure to outdoor fine particulate matter. Proc. Natl. Acad. Sci. USA.

[3] Yang B.Y., Guo Y., Markevych I., Qian Z.M., Bloom M.S., Heinrich J., Dharmage S.C., Rolling C.A., Jordan S.S., Komppula M. (2019). Association of Long-term Exposure to Ambient Air Pollutants with Risk Factors for Cardiovascular Disease in China. JAMA Netw. Open.

[4] Zheng H., Kong S., Chen N., Yan Y., Liu D., Zhu B., Xu K., Cao W., Ding Q., Lan B. (2020). Significant changes in the chemical compositions and sources of PM2.5 in Wuhan since the city lockdown as COVID-19. Sci. Total. Environ..

[5] Chen K., Wang M., Huang C., Kinney P.L., Anastas P.T. (2020). Air pollution reduction and mortality benefit during the COVID-19 outbreak in China. Lancet Planet. Health.

[6] Otmani A., Benchrif A., Tahri M., Bounakhla M., Chakir E.M., El Bouch M., Krombi M. (2020). Impact of Covid-19 lockdown on PM10, SO2 and NO2 concentrations in Salé City (Morocco). Sci. Total. Environ..

[7] Kanniah K.D., Zaman N.A.F.K., Kaskaoutis D.G., Latif M.T. (2020). COVID-19’s impact on the atmospheric environment in the Southeast Asia region. Sci. Total. Environ..

[8] Gautam S. (2020). The Influence of COVID-19 on Air Quality in India: A Boon or Inutile. Bull. Environ. Contam. Toxicol..

[9] Nakada L.Y.K., Urban R.C. (2020). COVID-19 pandemic: Impacts on the air quality during the partial lockdown in São Paulo state, Brazil. Sci. Total. Environ..

[10] Son J.-Y., Fong K.C., Heo S., Kim H., Lim C.C., Bell M.L. (2020). Reductions in mortality resulting from reduced air pollution levels due to COVID-19 mitigation measures. Sci. Total. Environ..

[11] Tobías A., Carnerero C., Reche C., Massagué J., Via M., Minguillón M.C., Alastuey A., Querol X. (2020). Changes in air quality during the lockdown in Barcelona (Spain) one month into the SARS-CoV-2 epidemic. Sci. Total Environ..

[12] Le Quéré C., Jackson R.B., Jones M.W., Smith A.J.P., Abernethy S., Andrew R.M., De-Gol A.J., Willis D.R., Shan Y., Canadell J.G. (2020). Temporary reduction in daily global CO2 emissions during the COVID-19 forced confinement. Nat. Clim. Chang..

[13] Boccia S., Ricciardi W., Ioannidis J.P.A. (2020). What Other Countries Can Learn From Italy During the COVID-19 Pandemic. JAMA Intern. Med..

[14] Alicandro G., Remuzzi G., La Vecchia C. (2020). Italy’s first wave of the COVID-19 pandemic has ended: No excess mortality in May, 2020. Lancet.

[15] Collivignarelli M.C., Abbà A., Bertanza G., Pedrazzani R., Ricciardi P., Miino M.C. (2020). Lockdown for CoViD-2019 in Milan: What are the effects on air quality?. Sci. Total. Environ..

[16] Chauhan A., Singh R.P. (2020). Decline in PM2.5 concentrations over major cities around the world associated with COVID-19. Environ. Res..

[17] Martelletti L., Martelletti P. (2020). Air Pollution and the Novel Covid-19 Disease: A Putative Disease Risk Factor. SN Compr. Clin. Med..

[18] Adhikari A., Yin J. (2020). Short-Term Effects of Ambient Ozone, PM(2.5,) and Meteorological Factors on COVID-19 Con-firmed Cases and Deaths in Queens, New York. Int. J. Environ. Res. Public Health.

[19] Bashir M.F., Jiang B., Komal B., Bashir M.A., Farooq T.H., Iqbal N., Bashir M. (2020). Correlation between environmental pollution indicators and COVID-19 pandemic: A brief study in Cal-ifornian context. Environ. Res..

[20] Liang D., Shi L., Zhao J., Liu P., Sarnat J.A., Gao S., Schwartz J., Liu Y., Ebelt S.T., Scovronick N. (2020). Urban Air Pollution May Enhance COVID-19 Case-Fatality and Mortality Rates in the United States. Innovation.

[21] Gill J.R., DeJoseph M.E. (2020). The Importance of Proper Death Certification during the COVID-19 Pandemic. JAMA.

[22] Raymond E., Thieblemont C., Alran S., Faivre S. (2020). Impact of the COVID-19 Outbreak on the Management of Patients with Cancer. Target. Oncol..

[23] Liang W., Guan W., Chen R., Wang W., Li J., Xu K., Li C., Ai Q., Lu W., Liang H. (2020). Cancer patients in SARS-CoV-2 infection: A nationwide analysis in China. Lancet Oncol..

[24] Rosenbaum L. (2020). The Untold Toll—The Pandemic’s Effects on Patients without Covid-19. N. Engl. J. Med..

[25] Zylke J.W., Bauchner H. (2020). Mortality and morbidity: The measure of a pandemic. JAMA.

[26] Coker E.S., Cavalli L., Fabrizi E., Guastella G., Lippo E., Parisi M.L., Pontarollo N., Rizzati M., Varacca A., Vergalli S. (2020). The Effects of Air Pollution on COVID-19 Related Mortality in Northern Italy. Environ. Resour. Econ..

[27] Scortichini M., Dos Santos R.S., Donato F.D., De Sario M., Michelozzi P., Davoli M., Masselot P., Sera F., Gasparrini A. (2020). Excess mortality during the COVID-19 outbreak in Italy: A two-stage interrupted time-series analysis. Int. J. Epidemiol..

[28] Liberti A. (1975). Modern methods for air pollution monitoring. Pure Appl. Chem..

[29] Powell H., Krall J.R., Wang Y., Bell M.L., Peng R.D. (2015). Ambient Coarse Particulate Matter and Hospital Admissions in the Medicare Cohort Air Pollution Study, 1999–2010. Environ. Health Perspect..

[30] Cai J. (2019). Humidity: Calculate Water Vapor Measures from Temperature and Dew Point.

[31] Armstrong B.G., Gasparrini A., Tobías A. (2014). Conditional Poisson models: A flexible alternative to conditional logistic case cross-over analysis. BMC Med. Res. Methodol..

[32] Gasparrini A., Guo Y., Hashizume M., Lavigne E., Zanobetti A., Schwartz J., Tobias A., Tong S., Rocklöv J., Forsberg B. (2015). Mortality risk attributable to high and low ambient temperature: A multicountry observational study. Lancet.

[33] Lian T., Fu Y., Sun M., Yin M., Zhang Y., Huang L., Huang J., Xu Z., Mao C., Ni J. (2020). Effect of temperature on accidental human mortality: A time-series analysis in Shenzhen, Guangdong Province in China. Sci. Rep..

[34] Gasparrini A., Leone M. (2014). Attributable risk from distributed lag models. BMC Med. Res. Methodol..

[35] Turner H., Firth D. (2020). Generalized Nonlinear Models in R: An Overview of the Gnm Package.

[36] Viechtbauer W. (2010). Conducting meta-analyses in R with the metafor package. J. Stat. Softw..

[37] Guo Y., Li S., Tian Z., Pan X., Zhang J., Williams G. (2013). The burden of air pollution on years of life lost in Beijing, China, 2004–2008: Retrospective regression analysis of daily deaths. BMJ.

[38] Dai L., Zanobetti A., Koutrakis P., Schwartz J.D. (2014). Associations of Fine Particulate Matter Species with Mortality in the United States: A Multicity Time-Series Analysis. Environ. Health Perspect..

[39] Liu C., Chen R., Sera F., Vicedo-Cabrera A.M., Guo Y., Tong S., Coelho M.S.Z.S., Saldiva P.H.N., Lavigne E., Matus P. (2019). Ambient Particulate Air Pollution and Daily Mortality in 652 Cities. N. Engl. J. Med..

[40] Zhang Y., Fang J., Mao F., Ding Z., Xiang Q., Wang W. (2020). Age- and season-specific effects of ambient particles (PM(1), PM(2.5), and PM(10)) on daily emergency de-partment visits among two Chinese metropolitan populations. Chemosphere.

[41] Zhu Y., Xie J., Huang F., Cao L. (2020). Association between short-term exposure to air pollution and COVID-19 infection: Evidence from China. Sci. Total Environ..

[42] Fattorini D., Regoli F. (2020). Role of the chronic air pollution levels in the Covid-19 outbreak risk in Italy. Environ. Pollut..

[43] Cole M.A., Ozgen C., Strobl E. (2020). Air Pollution Exposure and Covid-19 in Dutch Municipalities. Environ. Resour. Econ..

[44] Xiao W., Nethery R.C., Sabath B.M., Braun D., Dominici F. (2020). Exposure to air pollution and COVID-19 mortality in the United States: A nationwide cross-sectional study. medRxiv.

[45] Vasquez-Apestegui V., Parras-Garrido E., Tapia V., Paz-Aparicio V.M., Rojas J.P., Sánchez-Ccoyllo O.R., Gonzales G.F. (2020). Association between Air Pollution in Lima and the High Incidence of COVID-19: Findings from a Post Hoc Analysis. Res. Sq..

[46] Jiang Y., Wu X.-J., Guan Y.-J. (2020). Effect of ambient air pollutants and meteorological variables on COVID-19 incidence. Infect. Control. Hosp. Epidemiol..

[47] Yao Y., Pan J., Wang W., Liu Z., Kan H., Qiu Y., Meng X., Wang W. (2020). Association of particulate matter pollution and case fatality rate of COVID-19 in 49 Chinese cities. Sci. Total. Environ..

[48] Comunian S., Dongo D., Milani C., Palestini P. (2020). Air Pollution and COVID-19: The Role of Particulate Matter in the Spread and Increase of COVID-19’s Morbidity and Mortality. Int. J. Environ. Res. Public Health.

[49] Zhou F., Yu T., Du R., Fan G., Liu Y., Liu Z., Xiang J., Wang Y., Song B., Gu X. (2020). Clinical course and risk factors for mortality of adult inpatients with COVID-19 in Wuhan, China: A retrospective cohort study. Lancet.

[50] Van Doremalen N., Bushmaker T., Morris D.H., Holbrook M.G., Gamble A., Williamson B.N., Tamin A., Harcourt J.L., Thornburg N.J., Gerber S.I. (2020). Aerosol and Surface Stability of SARS-CoV-2 as Compared with SARS-CoV-1. N. Engl. J. Med..

[51] Wei M., Liu H., Chen J., Xu C., Li J., Xu P., Sun Z. (2020). Effects of aerosol pollution on PM2.5-associated bacteria in typical inland and coastal cities of northern China during the winter heating season. Environ. Pollut..

[52] Farina F., Sancini G., Battaglia C., Tinaglia V., Mantecca P., Camatini M., Palestini P. (2013). Milano Summer Particulate Matter (PM10) Triggers Lung Inflammation and Extra Pulmonary Adverse Events in Mice. PLoS ONE.

[53] Li B., Yang J., Zhao F., Zhi L., Wang X., Liu L., Bi Z., Zhao Y. (2020). Prevalence and impact of cardiovascular metabolic diseases on COVID-19 in China. Clin. Res. Cardiol..

[54] Ji Y., Ma Z., Peppelenbosch M.P., Pan Q. (2020). Potential association between COVID-19 mortality and health-care resource availability. Lancet Glob. Health.

[55] Rosenbaum L. (2020). Facing Covid-19 in Italy—Ethics, Logistics, and Therapeutics on the Epidemic’s Front Line. N. Engl. J. Med..

[56] Emanuel E.J., Persad G., Upshur R., Thome B., Parker M., Glickman A., Zhang C., Boyle C., Smith M., Phillips J.P. (2020). Fair Allocation of Scarce Medical Resources in the Time of Covid-19. N. Engl. J. Med..

[57] Barrett K., Khan Y.A., Mac S., Ximenes R., Naimark D.M., Sander B. (2020). Estimation of COVID-19–induced depletion of hospital resources in Ontario, Canada. Can. Med. Assoc. J..

[58] De Filippo O., D’Ascenzo F., Angelini F., Bocchino P.P., Conrotto F., Saglietto A., Secco G.G., Campo G., Gallone G., Verardi R. (2020). Reduced Rate of Hospital Admissions for ACS during Covid-19 Outbreak in Northern Italy. N. Engl. J. Med..

[59] De Rosa S., Spaccarotella C., Basso C., Calabrò M.P., Curcio A., Filardi P.P., Mancone M., Mercuro G., Muscoli S., Nodari S. (2020). Reduction of hospitalizations for myocardial infarction in Italy in the COVID-19 era. Eur. Hear. J..

[60] Sicard P., Khaniabadi Y.O., Perez S., Gualtieri M., De Marco A. (2019). Effect of O3, PM10 and PM2.5 on cardiovascular and respiratory diseases in cities of France, Iran and Italy. Environ. Sci. Pollut. Res..

[61] Zhao M., Wang M., Zhang J., Ye J., Xu Y., Wang Z., Ye D., Liu J., Wan J. (2020). Advances in the relationship between coronavirus infection and cardiovascular diseases. Biomed. Pharmacother..

[62] Kang S.-J., Jung S.I. (2020). Age-Related Morbidity and Mortality among Patients with COVID-19. Infect. Chemother..

[63] Han F., Yang X., Xu D., Wang Q., Xu D. (2019). Association between outdoor PM2.5 and prevalence of COPD: A systematic review and meta-analysis. Postgrad. Med. J..

[64] Yang Y., Qi J., Ruan Z., Yin P., Zhang S., Liu J., Liu Y., Li R., Wang L., Lin H. (2020). Changes in Life Expectancy of Respiratory Diseases from Attaining Daily PM2.5 Standard in China: A Nationwide Observational Study. Innovation.

[65] Hoek G., Kos G., Harrison R., de Hartog J., Meliefste K., ten Brink H., Katsouyanni K., Karakatsani A., Lianou M., Kotronarou A. (2008). Indoor–outdoor relationships of particle number and mass in four European cities. Atmos. Environ..

[66] Szigeti T., Dunster C., Cattaneo A., Cavallo D., Spinazzè A., Saraga D.E., Sakellaris I.A., de Kluizenaar Y., Cornelissen E.J., Hänninen O. (2016). Oxidative potential and chemical composition of PM2. 5 in office buildings across Europe—The OFFICAIR study. Environ. Int..

[67] Ji W., Zhao B. (2015). Estimating Mortality Derived from Indoor Exposure to Particles of Outdoor Origin. PLoS ONE.

