# Supplementary material for: Vulnerability and Burden of All-Cause Mortality Associated with Particulate Air Pollution during COVID-19 Pandemic: A Nationwide Observed Study in Italy
Source: Toxics. 2021 Mar 15;9(3):56. doi: 10.3390/toxics9030056 (PMC8001417; doi:10.3390/toxics9030056)
Supplement: Supplementary file 1 [file toxics-09-00056-s001.pdf]

Supplementary Materials: Vulnerability and Burden of All-Cause Mortality Associated with Particulate Air Pollution during COVID-19 Pandemic: A Nationwide Observed Study in Italy

Tingting Ye, Rongbin Xu, Wenhua Yu, Zhaoyue Chen, Yuming Guo and Shanshan Li

Method S1. Calibration of ERA5 Temperature Data Against Weather Observations

1.1. Data Collection

We collected the ERA5 hourly surface (at 2 meters above the land surface) ambient temperature and ambient dew point temperature at 0.1° × 0.1° spatial resolution from January 1st, 2015 to May 31st, 2020 for Italy and its surrounding areas. This data was downloaded from the ERA5-Land hourly data (https://cds.climate.copernicus.eu/cdsapp#!/dataset/reanalysis-era5-land?tab=form).

To valid and calibrate the ERA5 data, we downloaded hourly ambient temperature and ambient dew point temperature observed by weather stations from the Integrated Surface Database (https://www.ncdc.noaa.gov/isd) hosted by National Oceanic and Atmospheric Administration of US. A total of 519 weather stations with hourly weather observations during 2015–2020 for Italy and its surrounding areas were used to validate the EAR5 hourly dataset (Figure S1). Hourly observations were transformed into daily observations by averaging all hourly observations within each day. Validation and calibration were based on a total of 692,002 daily observations. We did not use the daily temperature observations from weather stations for our analyses mainly for two reasons: 1) the spatial coverage of those stations is not satisfactory, with many provinces not covered; 2) the daily temperature observations from many weather stations were often incomplete. In contrast, the ERA5 data has perfect spatial and temporal coverage, despite its less accuracy.

To improve the performance of the calibration model, we also collected data on elevation and leaf area index (LAI) that may affect temperatures. LAI is an indicator for the density of vegetation cover. The monthly LAIs for both high and low vegetation at 0.1° × 0.1° spatial resolution were downloaded from ERA5-Land monthly averaged data (https://cds.climate.copernicus.eu/cdsapp#!/dataset/reanalysis-era5-land-monthly-means?tab=form). The global elevation data at 7.5 arc second (i.e., 7.5/3600 degree × 7.5/3600 degree, about 250 m × 250 m) spatial resolution was downloaded from the NASA Shuttle Radar Topographic Mission (SRTM http://srtm.csi.cgiar.org/srtmdata/). We aggregated the elevation data to 0.1° × 0.1° spatial resolution by averaging elevation values of the 48 × 48 blocks of 7.5/3600 degree × 7.5/3600 degree pixels. ERA5 daily temperature, ERA5 LAI, and elevation data were linked to all weather stations according to date, longitude and latitude.

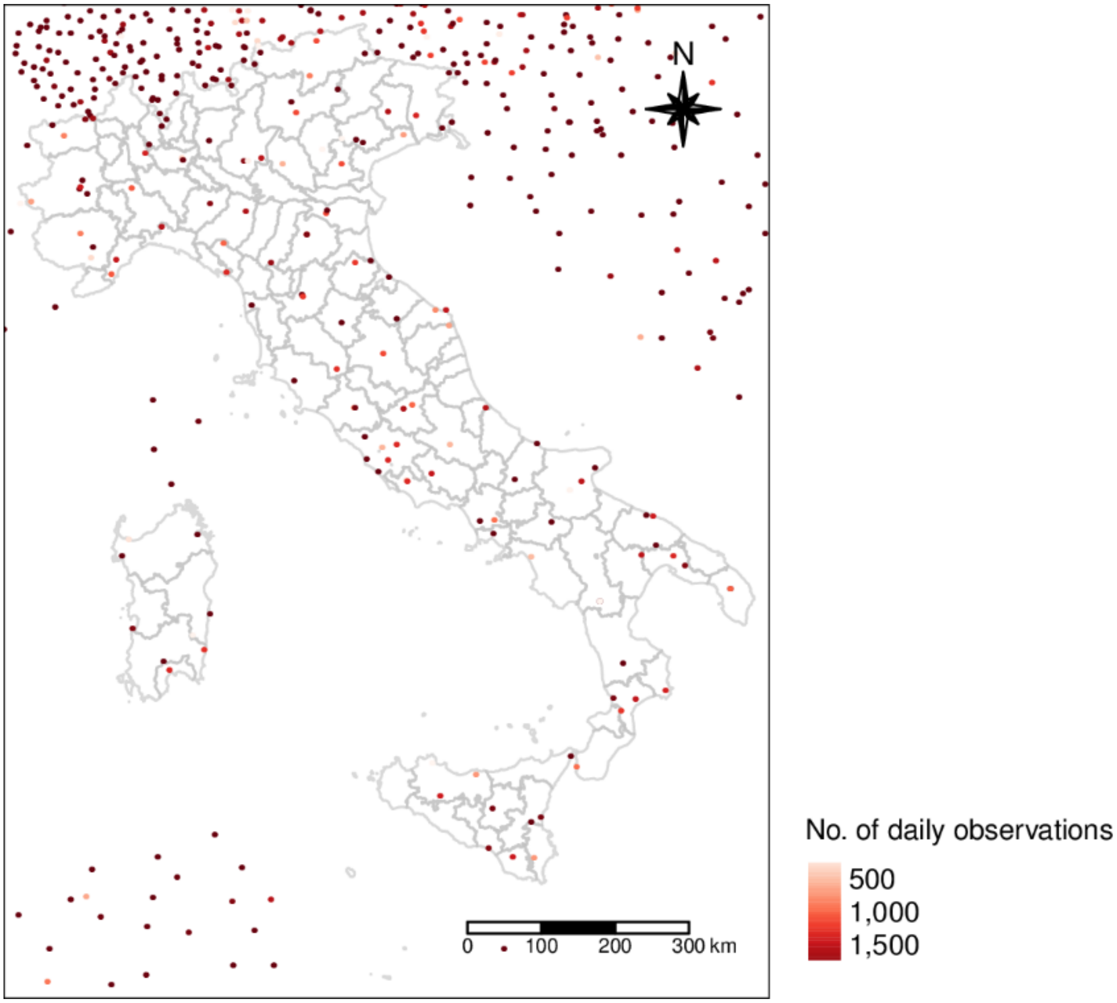

Figure S1. Distribution of weather stations that used to calibrate the ERA5 daily temperature and dew point temperature.

1.2. Calibration Model

We used random forest model to calibrate the ERA5 daily mean temperature and dew point temperature against the observations from weather stations. In the random forest models, the dependent variable was either the daily mean temperature or daily mean dew point temperature, and the independent variables (predictors) included ERA5 daily mean temperature, ERA5 daily mean dew point temperature, monthly LAI, elevation, year, day of year (1 to 366), latitude, longitude. The random forest models were fitted by the "ranger" (version 0.10.1) R package with default settings. The models built based on all the 692,002 daily observations from 519 weather stations were then used to calibrate the ERA5 daily temperature observations. The calibrated ERA5 daily mean temperature and ERA5 daily mean dew point temperature were then used to calculate daily mean relative humidity, using the algorithm provided by the "humidity" R package.

To evaluate the accuracy of the calibration model, we performed a 10-fold cross-validation by randomly splitting the 519 stations into 10 folds. As showed in Figure S2, the calibrated ERA5 temperatures (i.e., predicted values by the calibration model) were highly correlated with the observed values from weather stations, suggesting a high accuracy of the calibration model.

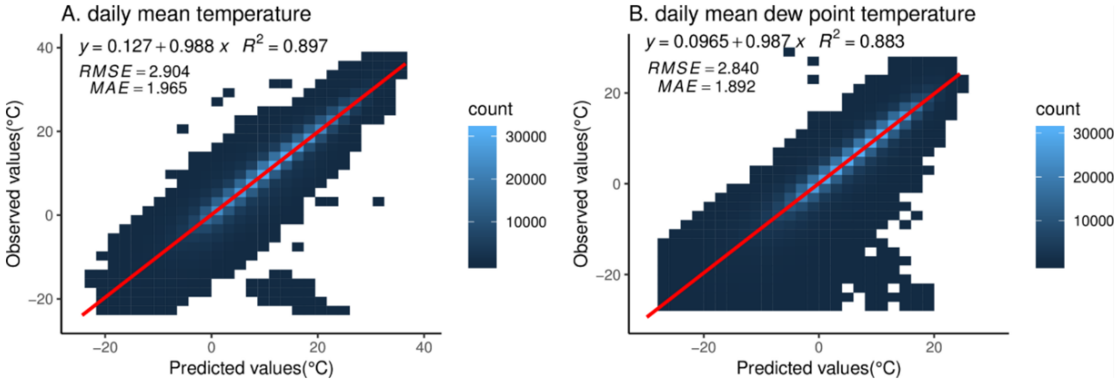

Figure S2. 10-fold cross-validation of the random forest model that using ERA5 daily mean temperature and dew point temperature and other covariates to predict the daily mean temperature and dew point temperature observed by weather stations. (A) daily mean temperature, and (B) daily mean dew point temperature.

Notes: other covariates included elevation, year, day of year (1 to 366), latitude, longitude, and leaf area index. We performed a 10-fold cross-validation by randomly splitting the 519 stations into 10 folds. RMSE, root mean square error. MAE, mean absolute error.

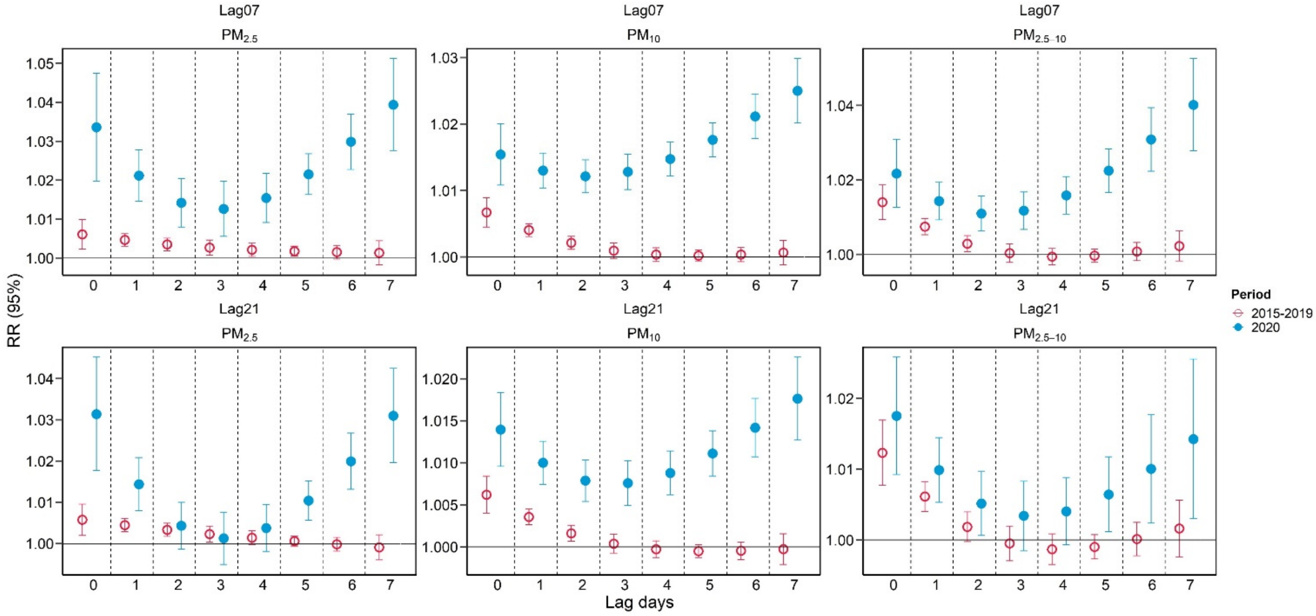

Figure S3. RR (mean and 95%CI) of all-cause mortality associated with per 10 μg/m³ increase in PM concentrations in single-pollutant models on different lag days of $PM_{2.5}$, $PM_{2.5}$ and $PM_{2.5\text{-}10}$.

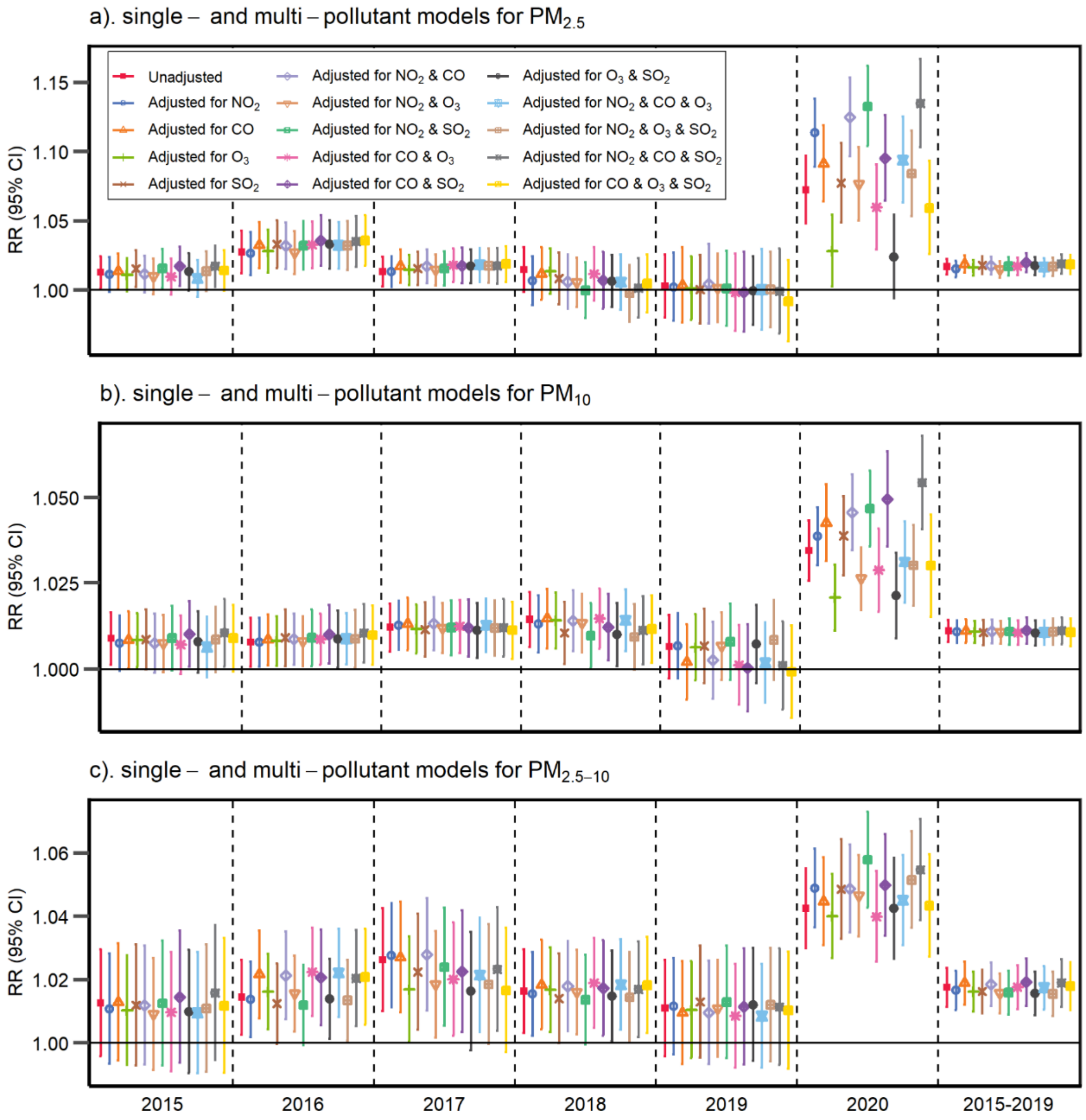

Figure S4. Cumulated RR (mean and 95%CI) of all-cause mortality associated with per 10 μg/m^3 increase in PM concentrations in single- and multi-pollutant models.

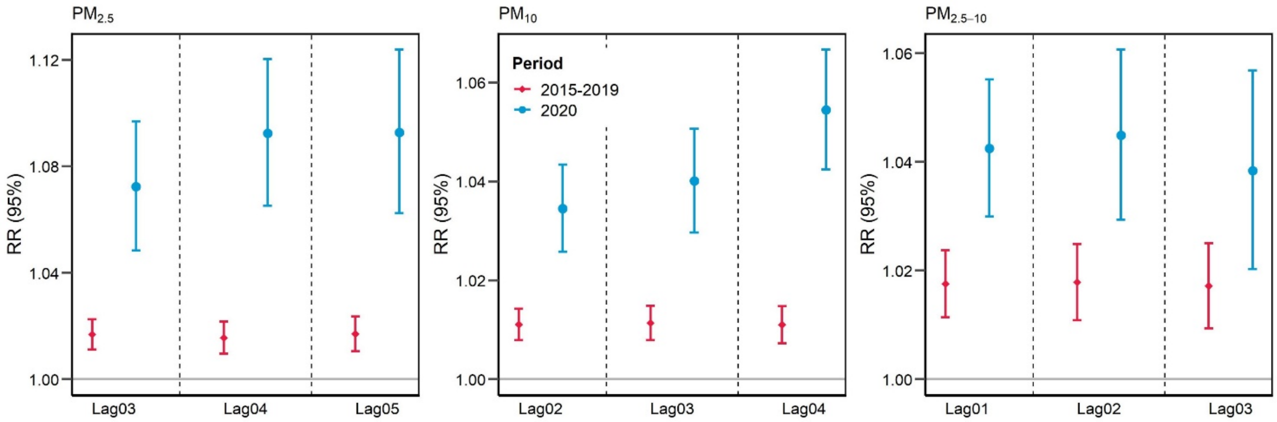

Figure S5. Cumulative RR (mean and 95%CI) of all-cause mortality associated with per 10 μg/m^3 increase in PM concentrations in single-pollutant models on different lag days of $PM_{2.5}$, $PM_{2.5}$ and $PM_{2.5\text{-}10}$.

Table S1. Estimation of the differences of increased relative risk (RR) between different time periods and subgroups.

		2015–2019		2020		2020 vs. 2015–2019
		**Increased RR (%) (95%CI)**	***p*-value***	**Increased RR (%) (95%CI)**	***p*-value***	***p*-value#**
$PM_{2.5}$	Total	1.69 (1.12, 2.25)	NA	7.24 (4.84, 9.70)	NA	<0.001
	Age < 65 years	0.00 (−1.67, 1.71)	reference	5.33 (−0.25, 11.22)	reference	0.07
	Age ≥ 65 years	1.88 (1.29, 2.49)	0.04	7.41 (4.92, 9.97)	0.52	<0.001
	Female	2.15 (1.38, 2.93)	reference	5.68 (2.87, 8.57)	reference	0.01
	Male	1.17 (0.36, 1.98)	0.09	8.81 (5.76, 11.93)	0.14	<0.001
PM_{10}	Total	1.11 (0.79, 1.42)	NA	3.45 (2.58, 4.34)	NA	<0.001
	Age < 65 years	0.13 (−0.80, 1.07)	reference	2.36 (0.19, 4.58)	reference	0.06
	Age ≥ 65 years	1.23 (0.89, 1.56)	0.03	3.56 (2.65, 4.47)	0.33	<0.001
	Female	1.41 (0.98, 1.85)	reference	3.63 (2.57, 4.69)	reference	<0.001
	Male	0.77 (0.32, 1.22)	0.04	3.27 (2.15, 4.39)	0.64	<0.001
$PM_{2.5-10}$	Total	1.76 (1.14, 2.38)	NA	4.25 (2.99, 5.51)	NA	<0.001
	Age < 65 years	1.32 (−0.52, 3.20)	reference	3.13 (0.22, 6.13)	reference	0.31
	Age ≥ 65 years	1.81 (1.16, 2.46)	0.63	4.36 (3.06, 5.68)	0.46	<0.001
	Female	2.09 (1.25, 2.94)	reference	4.56 (3.08, 6.06)	reference	<0.001
	Male	1.39 (0.51, 2.28)	0.26	3.93 (2.40, 5.50)	0.57	0.004

Note: *p*-value was estimated by fixed effect meta-regression. * *p*-value for differences in subgroups; # *p*-value for differences in different time periods where the normal period of 2015-2019 was taken as the reference group.
